# Surgical treatment of gastric GIST with acute bleeding using laparoscopic sleeve gastrectomy: A report of two cases

**DOI:** 10.1002/ccr3.2093

**Published:** 2019-03-12

**Authors:** Nicola Chetta, Arcangelo Picciariello, Carlo Nagliati, Alessandro Balani, Gennaro Martines

**Affiliations:** ^1^ General Surgery “M.Rubino” Azienda Ospedaliero Universitaria Policlinico Bari Italy; ^2^ Dipartimento Chirurgico Ospedale di Gorizia Gorizia Italy

**Keywords:** gastric bleeding, GIST, laparoscopy, sleeve gastrectomy

## Abstract

In this report, we want to emphasize how a laparoscopic bariatric surgical procedure, in experienced hands, has shown to be a valid alternative for the hemorrhage control and the removal of a gastrointestinal tumor in a life‐threatening situation.

## 1 INTRODUCTION

Gastrointestinal stromal tumors (GISTs) are the most common mesenchymal tumors of the gastrointestinal tract. They arise from the Interstitial cells of Cajal (ICCs) that are implicated in the regulation of gut peristalsis, with a pacemaker role. GISTs are more often located in the stomach (56%) although they can arise in any portion of the digestive tract.^1^ Clinical manifestations depend on the size and the site of the primary lesion. Most of GISTs are asymptomatic and thus incidentally discovered during endoscopic, imaging, or surgical procedures performed for unrelated complaints. Affected patients generally seek medical assistance due to symptoms resulting from either ulceration or mass effects. Typically, the most frequent manifestation is gastrointestinal bleeding which may be associated with anemia and melena or hematemesis, often requiring urgent surgical intervention.^2^ Recent data suggest that gastrointestinal bleeding is caused by tumor invasion of the mucosa layer, resulting in ulceration. Many studies have reported that the prognosis of GISTs with gastrointestinal bleeding is relatively poor compared with patients without bleeding and that GISTs’ bleeding may be an independent risk factor for recurrence.^3^

Although the treatment of gastrointestinal stromal tumors with tyrosine kinase inhibitors is one of the best examples of the advances of molecular targeted therapy over the last two decades, surgical resection with clear margins remains the mainstay of cure.^4^ At present, the laparoscopic approach can be seen as a valid alternative to traditional surgery in the treatment of GISTs, and a feasible and secure method if performed by an experienced surgeon in properly selected patients.

Herein, we report the cases of two patients from two different Italian hospitals who have undergone a successful laparoscopic sleeve gastrectomy in emergency following the acute bleeding of a gastric GIST.

Laparoscopic sleeve gastrectomy (LSG) is the most commonly carried out bariatric surgical procedure worldwide; by removing approximately 75% of the stomach from the greater curvature side, it acts as a restrictive procedure also capable of changes in the secretion of gastrointestinal hormones involved in the weight loss.^5^ The incidence of GIST is suspected to be more in obese patients undergoing bariatric surgery (0.6%‐0.8%) in comparison with the general population (0.0006%‐0.0015%). The bariatric surgeon has to inspect the stomach during laparoscopy for such tumors and manage the incidentally encountered during a laparoscopic bariatric operation.^6^ This is particularly true during gastric bypass, when undiagnosed disease may remain in the excluded stomach, which will be inaccessible to endoscopic exams but also during the LSG preparation, when a possible lesion could be included in the staple line or not removed at all.

In this report, we present two cases in which a gastric GIST is not an incidental finding during a laparoscopic bariatric operation but a laparoscopic bariatric surgical procedure, in experienced hands, has shown to be a valid alternative for the hemorrhage control and the removal of a gastrointestinal tumor in a life‐threatening situation.

## 2 CASE 1

**Figure 1:**
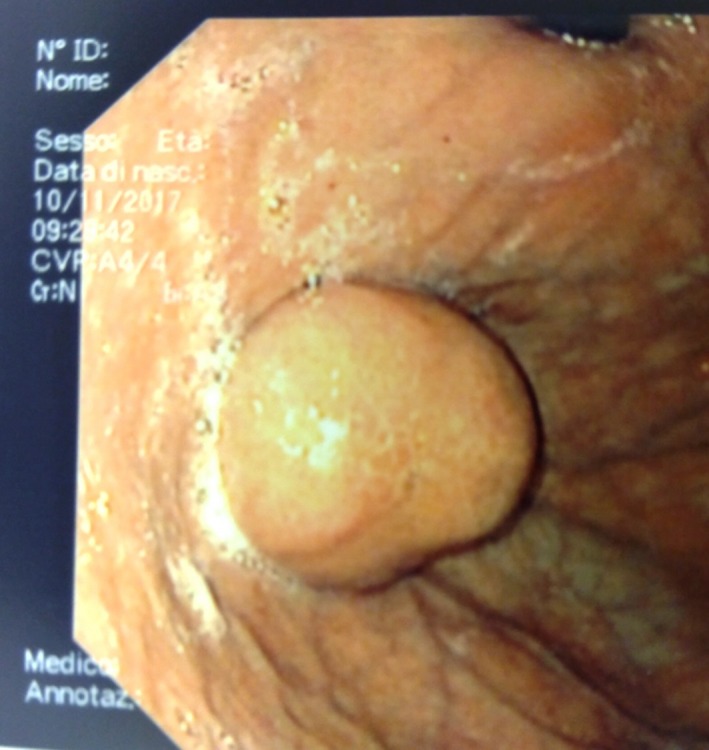
Endoscopic view: gastric submucosal neoplasm of the greater curvature at the gastric fundus

**Figure 2:**
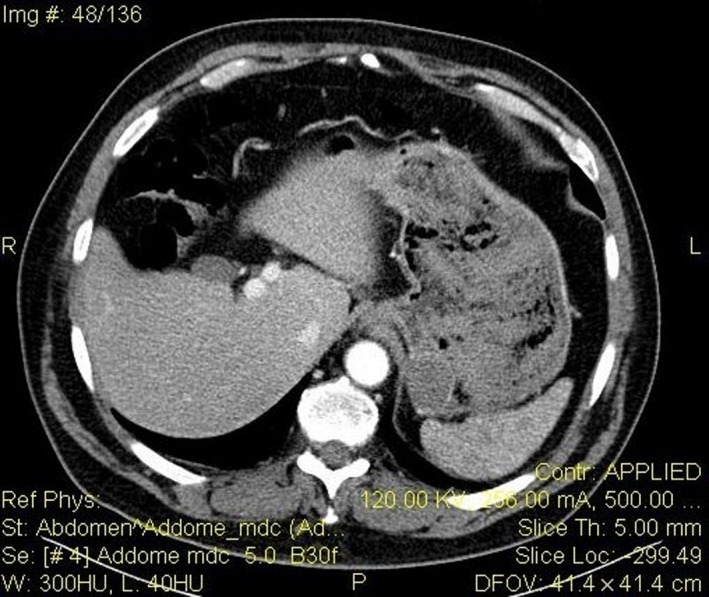
TC abdomen with medium contrast: neoplasm of the gastric fundus. No evidence of bleeding

A 67‐year‐old man presented himself in our emergency department after having several episodes of hematemesis and melena associated with chest pain over the previous 24 hours. The patient had a known history of gastric ulcer developed 20 years before without any other comorbidities except for class II obesity (BMI 36.68 kg/m^2^). Electrocardiogram (ECG) and serum troponin levels excluded an ischemic cardiac event, but the results for complete blood cell counts and hemocoagulation revealed a hemoglobin value of 9.6 g/dL. Two days before, the patient had undergone an esophagogastroduodenoscopy (OGD; Figure 1) for a recurrent digestive discomfort after eating, and he was found to have a 30‐mm submucosal lesion in the posterior wall of the greater curvature at the gastric fundus that was biopsied. The following OGD performed in urgency identified the origin of bleeding at the biopsy site; a temporary hemostasis was attempted using epinephrine injection therapy. In addition, an abdominopelvic computed tomography (CT) with enhanced scans was performed to check for any further bleeding, with negative result (Figure 2). Nevertheless, 12 hours following endoscopy the patient had another melena accompanied by an episode of loss of consciousness; the hemoglobin level was 6.9 g/dL. Based on the recurrence of the gastric bleeding, the patient was prepared for surgery.

**Figure 3:**
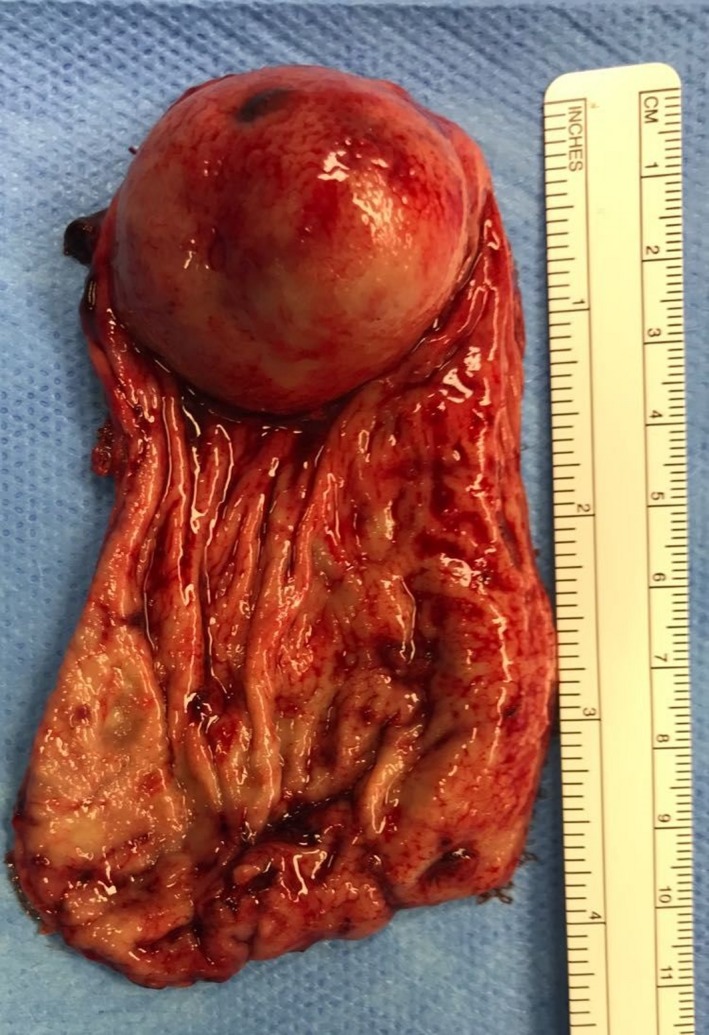
Gastric specimen: polypoid mass with hemorrhagic foci 40 × 35 mm in size

Diagnostic laparoscopy was performed to further evaluate the cause of the acute abdomen revealing only distension of the stomach, with no evidence of exophytic lesion, and ruled out signs of distant disease. An intraoperative endoscopy with a standard endoscope (Olympus GIF‐Q‐165®) was used to aspirate and lavage the stomach as well as confirming the localization of the bleeding lesion in the greater curvature at the gastric fundus. Subsequently, a laparoscopic sleeve gastrectomy (LSG) was carried out. Five trocars have been placed in the upper abdominal quadrants: one 12‐mm trocar above the umbilicus in the midline, three 12‐mm trocars in right subcostal, subxiphoid, and left subcostal, and one 5‐mm trocar in the left anterior axillary line. The gastric resection was performed using a linear stapler (Echelon Flex™ 60 Endopath®) applied alongside a 38‐Fr bougie. The next step has been a methylene blue dye test in order to check the sealing of the staple line that was additionally reinforced by nebulization with cyanoacrylate sealant (Glubran 2®) used also to create an adhesion with the greater omentum as a chemical omentoplasty.^7^ The surgical gastrectomy specimen (Figure 3) was retrieved through the slightly enlarged left subcostal access. A drain was placed at the end of the procedure. The patient was administered 3 units of packed red cells to stabilize his hemoglobin up to 9.7 g/dL. Operative time has been 92 minutes.

The patient's postoperative course was uneventful, except for a left subcostal wound infection which was opened and drained at the bedside and for a *E coli* urinary tract infection which was successfully treated with antibiotics. The diagnosis of gastric GIST was confirmed on the surgical specimen that showed a polypoid mass with hemorrhagic foci 40 × 35 mm in size; surgical resection margins were negative (R0). The mitotic rate was <1/50 HP; CD34, CD 117/C‐kit, and smooth muscle actin were positive; the Ki67‐MIB1 immunostaining indicated a low proliferative rate (count rate 4%). The tumor was staged as GIST of gastric origin in the very low‐risk category so that a systemic adjuvant chemotherapy was not needed^8^ Oral nutrition commenced on postoperative day 6, after a Gastrografin® swallow that confirmed a regular gastric transit and the absence of leaks. The patient was discharged on the 8th postoperative day. At one‐month postoperative follow‐up, no discomfort on eating or other symptoms were referred; he had lost 15 kg from the day of the operation (BMI 29.41 kg/m^2^).

## 3 CASE 2

**Figure 4:**
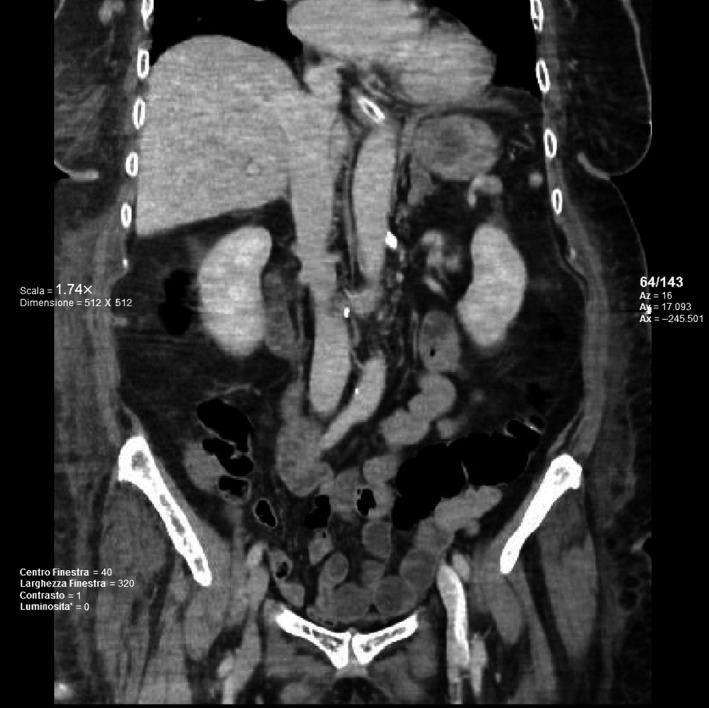
Coronal TC abdomen neoplasm of the gastric fundus

A 83‐year‐old female patient was referred to the intensive care unit (ICU) in emergency state for the acute onset of a respiratory failure accompanied by high‐frequency atrial fibrillation (AF). She had a body mass index (BMI) of 29.52 kg/m^2^ and a past medical history of hypertension, hyperthyroidism, and atrial fibrillation (AF) under treatment with direct oral anticoagulants (DOACs). The patient also had a history of previous surgery represented by a laparotomic left hemicolectomy for a benignant colonic stenosis. She was initially managed with intubation and received mechanical ventilation, and once hemodynamic stability was achieved, her conditions worsened due to unexpected upper gastrointestinal bleeding (UGIB). She was given a blood transfusion of 4 units of red blood cells, but her hemoglobin value continued to stay under 9.0 g/dL. The following OGD showed an ulcerated submucosal lesion of 3 cm at the greater curvature of the gastric fundus (Figure 4). Abdominopelvic computed tomography (CT) with enhanced scans revealed the presence of a solid mass with a soft contour enhanced by the intravenous contrast at the gastric fundus 4.5 × 4.5 cm in size (Figure 4).

**Figure 5:**
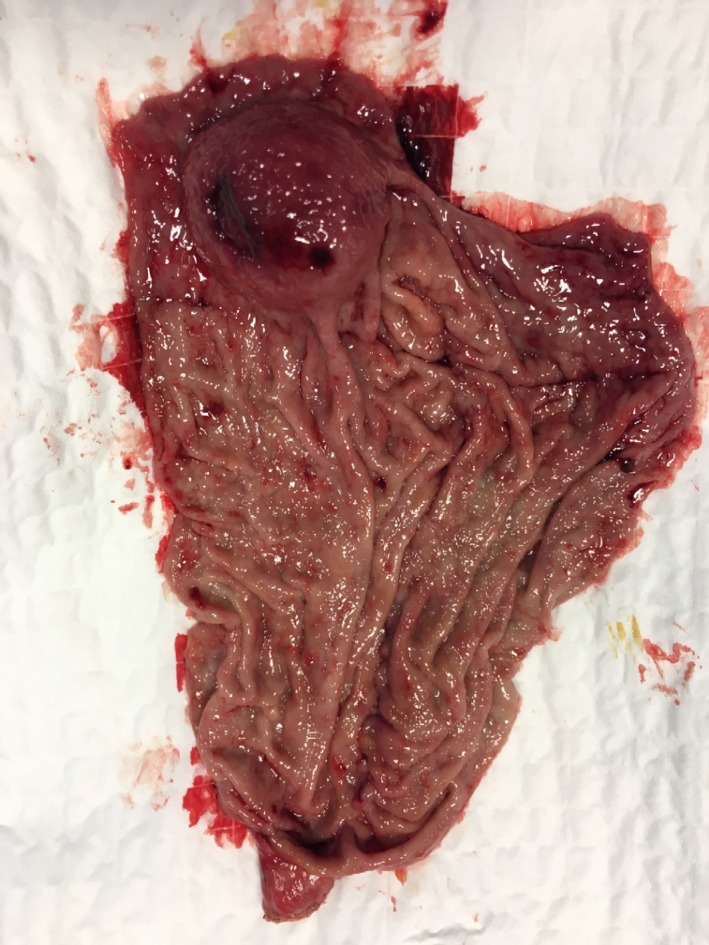
Gastric specimen: polypoid mass 30 mm in size

In the management of this acute situation in order to avoid any further delay of surgical intervention, the patient was prepared for an emergency laparoscopy. Four trocars have been placed in the upper abdominal quadrants: one 12‐mm trocar above the umbilicus in the midline, two 12‐mm trocars in right and left subcostal, and one 5‐mm trocar in the right anterior axillary line. The exploration of the abdomen cavity showed the adhesions attributable to the previous lower abdominal surgery as well as the cirrhotic changes of the parenchymal status of the liver. Once the stomach has been exposed, a nodule appeared on its surface near the greater curvature at the fundus. Subsequently, a laparoscopic sleeve gastrectomy (LSG) was carried out. The gastric resection was performed using a linear stapler (Echelon Flex™ 60 Endopath®) applied alongside a 38‐Fr bougie. The gastric specimen (Figure 5) was extracted through the epigastric access. One Jackson‐Pratt drainage tube was positioned on the resected gastric surface. Upper gastrointestinal contrast (Gastrografin®) was performed on the fifth postoperative day. Afterward, the patient was put on a liquid diet and discharged from the intensive care unit on the seventh postoperative day. The histopathology report of the gastric specimen showed a polypoid submucosal mass of 3 cm with smooth margins and a normal overlying mucosa with a central ulceration; there was no margin involvement (R0). Immunohistochemistry indicated positivity for CD34, CD 117/C‐kit, and smooth muscle actin; the mitotic count was <5/50 HP, and ki67‐MIB1 revealed a low proliferative rate. These findings were compatible with a gastric GIST in the low‐risk category.

The postoperative period was uneventful for complications except for the incidental finding of a lump in the patient's right breast The lesion was biopsied, and she was found to have an invasive ductal carcinoma for whom she was referred to the hospital's breast unit after discharge. The patient was discharged on the 15th postoperative day. She was subsequently seen at one month after surgery once resumed a normal diet with no reported symptoms.

## 4 DISCUSSION

Surgical resection with free margins of tumor disease (R0) is the only potentially curative option for GISTs. In the case of gastric location, which is the most common, based on the assumption that GISTs do not have the typical lymphotropism, infiltrating behavior and propensity to metastatize of adenocarcinomas, a less extensive surgery like in the case of wedge resections, is suitable to treat the disease and spare the stomach.^9^ Over the last two decades, certainly minimal invasive approaches for gastric GIST did not only become more popular in clinical routine but were supported by strong evidence from literature.^10^ Laparoscopy is advantageous due to the less need for pain medications, the earlier food intake, the reduced peri‐operative morbidity, and the shorter hospitalization if compared with open surgery. Limitations about the feasibility of laparoscopy in the surgical treatment of gastric GISTs can be related to the location and the size of the tumor. In the most recent guidelines of the European Society for Medical Oncology, the National Comprehensive Cancer Network (NCCN), and the Asian GIST guidelines, the indication for laparoscopic resection is reduced for lesions in the greater curvature and anterior wall of the gastric body, fundus, and antrum.^11^ For those of the lesser curvature, the cardia and the prepyloric region and the risks of stenosis of the lumen postoperatively or nononcological resections in addition to the difficulty in exposing the tumor affect negatively the choice of a laparoscopic approach. Concerning the limit of tumor's size, several retrospective cohort studies have suggested that laparoscopic resection is feasible and safe for gastric GISTs smaller than 5 cm. Likewise, the NCCN guidelines recommend laparoscopic resection for patients with gastric GISTs 5 cm or smaller.^12^ Nevertheless recent studies show the safety and feasibility of laparoscopic resection in large (>5 cm) and giant (>10 cm) GISTs with oncologic results comparable to those of open surgery.^13^ Other minimally invasive techniques such as submucosal tunneling endoscopic resection, endoscopic full thickness resection, and laparoscopic endoscopic cooperative surgery have recently shown good clinical outcomes; however, more studies and randomized clinical trials are necessary for a better definition of their long‐term safety.^8^ In contrast, open surgery remains an acceptable option in case of GISTs requiring complex multivisceral resection or large lesions that need delicate tissue handling to minimize the risk of tumor rupture and seeding especially when the specimen is removed.^14^ In writing about indications to laparoscopy in approaching gastric GISTs, it is of the utmost importance keep in mind their emergency presentations which often pose a challenge to the general surgeon both in terms of diagnosis and treatment. Some GISTs are therefore managed without a histological diagnosis. In the two cases reported, the diagnosis of GIST was unknown before surgery which has been performed prior for hemorrhage control.

In the Case 1, the patient was admitted to the emergency department with hematemesis and melena 48 hours following the endoscopic biopsy of a suspicious mass of the greater curvature at the gastric fundus, 30 mm in size. Conventional endoscopic forceps biopsy of GISTs is difficult and does not obtain enough tissue for a definitive diagnosis; EUS‐FNA (Endoscopic ultrasound‐guided‐fine needle aspiration biopsy) is the most established tissue sampling method.^1^ In both situations, endoscopists should pay special attention to intraoperative bleeding, perforation, or tumor cell seeding. In the Case 2, the upper gastrointestinal bleeding has been an unexpected event that worsened the conditions of a patient already admitted to the intensive care unit for cardiopulmonary complaints. The OGD showed an ulcerated submucosal lesion of 3 cm at the greater curvature of the gastric fundus whose bleeding was leading to hemorrhagic shock. In this critical setting, she was soon prepared for diagnostic laparoscopy while in the Case 1 the failure of endoscopic hemostasis preceded surgery.

In the Case 1, the exploration of the abdomen cavity showed no evidence of gastric exophytic lesions; an intraoperative endoscopy was useful in order to locate the mass and guide the chose of the proper resection. In the Case 2, the location of the tumor was well defined by the presence of a nodular lesion located on the greater curvature at the anterior wall of the gastric fundus.

In both cases, in light of the favorable location of the mass at the greater curvature, in the absence of distant disease, laparoscopic sleeve gastrectomy (LSG) has been chosen in order to treat the bleeding, remove the potential GIST, and spare the stomach calibrating the resection by the use of a 38‐Fr bougie placed against the lesser curvature. In addition, the fact that the procedure was carried out by experienced bariatric surgeons allowed to be more confident in facing emergency surgery with the use of a standardized laparoscopic technique, which reduced also operative time. Although LSG is considered an easier technique if compared with other bariatric procedure since no anastomosis is required, it includes some key technical points like the dissection of the stomach from the spleen, necessitating adequate laparoscopic expertise. Moreover in our cases, the presence of a blood‐filled stomach due to the hemorrhage may have had implications on the sealing of the staple line. In the Case 1, aerosolized cyanoacrylate sealant Glubran 2® has been used as a reinforcement of the staple line to prevent leak and bleeding.

The patients’ postoperative recovery was uneventful for major complications. Pathology reports showed in both cases complete surgical resection (R0); the tumors were diagnosed as gastric GISTs in the very low‐risk/low‐risk category, and adjuvant chemotherapy was not necessary.^15^ At one‐month postoperative follow‐up, no symptoms were referred by the patients. In the Case 1, the patient, who suffered also for morbid obesity, had lost 15 kg from the day of the operation (BMI 29.41 kg/m^2^). Wang Y et al reported their experience of laparoscopic sleeve gastrectomy in the treatment of GISTs in morbid obese patients. They found the LSG to be the best choice for obese patients combined with GISTs. It can remove tumors and excess stomach away at the same time.^16^ However, more studies, dedicated trials, and further confirmation are still needed before accepting this approach into the clinical practice.

## 5 CONCLUSIONS

Gastrointestinal stromal tumors (GISTs) are the most common mesenchymal tumors of the gastrointestinal tract. Surgical resection with free margins of tumor disease (R0) is their only curative option. In the case of potentially resectable gastric tumors, laparoscopic wedge resections are considered the standard therapy. This report describes the application in two cases of the laparoscopic sleeve gastrectomy for the emergency treatment of a bleeding gastric GIST. It can be concluded that a laparoscopic bariatric surgical procedure, in experienced hands, has shown to be a valid alternative for the hemorrhage control and the removal of a gastrointestinal tumor in a life‐threatening situation.

The importance of this surgical speciality has too often been underestimated, while the clinical, social, and economic benefits cannot be ignored.

## CONFLICT OF INTEREST

The authors declare that they have no competing interests.

Consent for publication: Written informed consent was obtained from the patients for publication of this case report and any accompanying images. A copy of the written consent is available for review by the Editor‐in‐Chief of this journal.

## AUTHORS CONTRIBUTION

NC: researched the data, wrote the manuscript, and contributed to the discussion. AP: wrote the manuscript, contributed to the discussion, and reviewed the manuscript. CN: performed one of the bariatric interventions. AB: contributed to the discussion and reviewed the manuscript. GM: wrote the manuscript, contributed to the discussion, and reviewed and edited the manuscript. All authors read and approved the final manuscript.
